# Ovarian Torsion Leading to Necrosis: A Case Report of Acute Abdominal Pain in a 25-Year-Old Female

**DOI:** 10.7759/cureus.67507

**Published:** 2024-08-22

**Authors:** Anjali Kumari, Gaurav V Mishra, Pratapsingh Parihar, Sakshi S Dudhe, Paritosh N Bhangale, Rakshanda Agrawal, Dhananjay Shinde

**Affiliations:** 1 Radiodiagnosis, Jawaharlal Nehru Medical College, Datta Meghe Institute of Higher Education and Research, Wardha, IND

**Keywords:** ultrasound imaging, gynecological emergency, ovarian cyst, laparoscopy, acute abdominal pain, ovarian torsion

## Abstract

Ovarian torsion is a critical gynecological emergency that presents with sudden-onset abdominal pain and requires immediate intervention to prevent irreversible ovarian damage. This case report describes a 25-year-old female who presented with acute right lower quadrant pain, which had escalated to excruciating levels over the past 45 minutes, accompanied by persistent nausea and vomiting. She had no fever, vaginal bleeding, or dysuria, and her urine pregnancy test was negative. A physical examination revealed significant tenderness and guarding in the right lower abdomen, with no evidence of organomegaly or abnormal pelvic findings. Imaging studies, including ultrasound, confirmed the diagnosis of a complete ovarian torsion with associated necrosis. The patient underwent successful laparoscopic surgery, which involved the removal of the necrotic ovary and affected fallopian tube. Postoperative recovery was uneventful, and the patient fully recovered within a week. This case underscores the importance of early diagnosis and surgical intervention in managing ovarian torsion to preserve ovarian function and prevent complications.

## Introduction

Ovarian torsion is a critical gynecological emergency characterized by the twisting of the ovary around its supporting ligaments, often leading to compromised blood flow and subsequent ischemia. This condition is most frequently associated with ovarian cysts or tumors, but it can also occur in the absence of any obvious pathology [1]. The incidence of ovarian torsion is estimated to be 2.7 to 7.4 cases per 100,000 women annually, with a peak incidence occurring between the ages of 20 and 40 [2]. The presentation of ovarian torsion can be insidious, with symptoms ranging from acute abdominal pain to nausea and vomiting. The pain is often sudden and localized to the lower abdomen or pelvis. Patients may also experience nausea, vomiting, and, less commonly, fever [3]. Due to the nonspecific nature of the symptoms, ovarian torsion can be challenging to diagnose, particularly in the absence of classic findings on physical examination and imaging studies [4].

Ultrasound imaging, often augmented with Doppler studies, is the primary diagnostic tool for ovarian torsion. Gray-scale ultrasound may reveal an enlarged, edematous ovary with or without an associated cyst, while Doppler studies can identify the absence of venous and arterial blood flow to the affected ovary [5]. Early diagnosis and intervention are crucial, as delayed treatment can result in ovarian necrosis and loss of ovarian function, with potential long-term implications for fertility [6]. Surgical intervention is the definitive treatment for ovarian torsion and typically involves laparoscopic detorsion and stabilization of the affected ovary. In cases where the ovary has undergone significant necrosis, oophorectomy may be necessary [7]. Prompt recognition and surgical management are essential to prevent adverse outcomes and preserve ovarian function.

## Case presentation

A 25-year-old female presented with acute abdominal pain localized to the right lower quadrant. She reported experiencing intermittent episodes of pain over the past several weeks, but in the last 45 minutes, the pain had become unbearable and excruciating. She also complained of persistent nausea and multiple episodes of vomiting. There was no history of fever, vaginal bleeding, discharge, dysuria, or changes in bowel habits. She denied any history of substance use. A urine pregnancy test (UPT) was negative.

On general examination, the patient appeared distressed and was clutching her right lower abdomen. Her vital signs were unstable, with a blood pressure of 145/90 mmHg and a pulse rate of 110 beats per minute. A gastrointestinal examination revealed tenderness and guarding in the right lower quadrant, with normal bowel sounds and no organomegaly. Pelvic examination demonstrated right adnexal fullness and significant pain on palpation but no evidence of bleeding or discharge.

**Figure 1:**
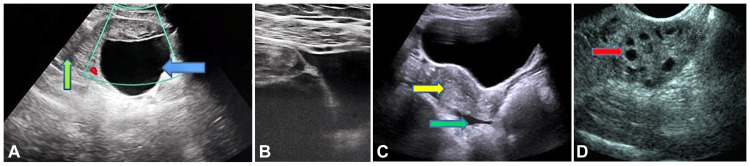
(A) A gray-scale B-mode ultrasound image revealed a bulky right ovary with peripherally arranged follicles measuring approximately 8.1 x 6.1 cm. A well-defined anechoic cystic lesion, measuring approximately 6.7 x 4.1 cm, was noted in the right ovary, with a thickened surrounding stroma appearing hypoechoic due to edema. The lesion showed no vascularity on color Doppler (blue arrow). There was evidence of minimal pericystic fluid collection and surrounding fat stranding (green arrow). These features are suggestive of complete ovarian torsion. (B) A gray-scale B-mode ultrasound image showed mild free fluid in the pelvis (white arrow). (C) A gray-scale B-mode ultrasound image depicted a normal uterus (yellow arrow) and mild fluid collection in the pouch of Douglas (green arrow). (D) A gray-scale B-mode ultrasound image of the left ovary showed a normal appearance with multiple follicles (red arrow).

**Figure 2:**
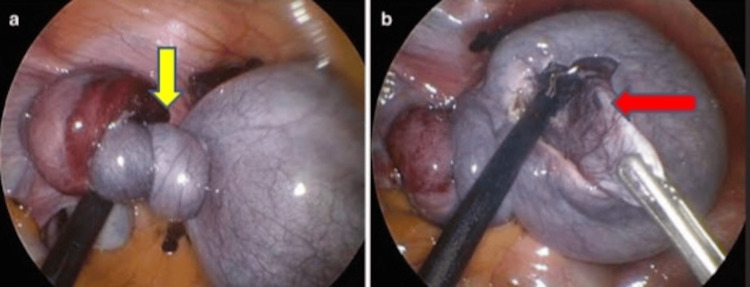
(A,B) Laparoscopic images showed the twisted right pedicle, containing the fallopian tube, ovarian ligaments, and ovarian vessels (yellow arrow). The red arrow in (B) highlights the bulky, edematous right ovary, which appeared bluish due to congestion and showed areas of necrosis due to broad ligament torsion.

The patient underwent surgical management via emergency laparoscopy. Intraoperative findings revealed a right ovarian cyst with complete torsion of the right ovary and fallopian tube, leading to necrosis. These findings were consistent with the ultrasound results depicted in Figure 1. The right fallopian tube and the ovarian ligaments were clamped, cut, ligated, and transfixed (Figure 2). The right ovary was removed, and specimens of these structures were collected. The procedure was uneventful, and the patient fully recovered within a week.

## Discussion

Ovarian torsion is a critical gynecological emergency characterized by the twisting of the ovary, which can lead to compromised blood flow and potential necrosis if not promptly addressed. The condition is typically associated with the presence of an ovarian cyst or mass, although it can occur in the absence of these findings [8,9]. This case highlights several key aspects of ovarian torsion, including its presentation, diagnostic approach, and management. The presentation of ovarian torsion can be insidious, often mimicking other abdominal conditions. The patient, in this case, reported acute, severe right lower quadrant pain, which had escalated in intensity over a short period. This rapid onset of severe pain, combined with nausea and vomiting, is consistent with the typical presentation of ovarian torsion [10]. The absence of fever, vaginal bleeding, or urinary symptoms can often lead to a delay in diagnosis, as these findings are not always present in cases of torsion [6].

Ultrasound imaging is a crucial diagnostic tool in suspected cases of ovarian torsion. In this patient, a gray-scale B-mode ultrasound revealed a bulky right ovary with a non-vascular cystic lesion and minimal pericystic fluid collection, which are typical signs of torsion [11]. The lack of blood flow on Doppler imaging further supported the diagnosis. Imaging studies are essential for distinguishing ovarian torsion from other abdominal conditions, such as appendicitis or ectopic pregnancy, which can present with similar symptoms [6]. Timely surgical intervention is critical in managing ovarian torsion. The primary goal of surgery is to restore blood flow to the affected ovary and, if necessary, to remove necrotic tissue. In this case, laparoscopic surgery was employed, allowing for direct visualization and correction of the torsion. The findings of a twisted pedicle and necrotic ovary corroborated the preoperative imaging results and underscored the importance of prompt surgical treatment [6]. Laparoscopy is the preferred approach for managing ovarian torsion due to its minimally invasive nature and effectiveness in both diagnosing and treating the condition [12]. In this case, the procedure was uneventful, and the patient recovered fully within a week, highlighting the positive outcomes associated with early intervention and appropriate surgical management.

## Conclusions

In conclusion, this case highlights the critical importance of prompt recognition and intervention in cases of ovarian torsion, a rare but serious gynecological emergency. The rapid escalation of symptoms and targeted imaging played a pivotal role in the timely diagnosis and management of this condition. Surgical intervention via laparoscopy confirmed the diagnosis and effectively treated the torsion, preventing further complications such as widespread necrosis and potential infertility. This case underscores the need for heightened clinical awareness of ovarian torsion in women presenting with acute lower abdominal pain, as early surgical management is essential for preserving ovarian function and ensuring favorable patient outcomes.
